# Intake of micronutrients and fatty acids of vegetarian, vegan, and omnivorous children (1–3 years) in Germany (VeChi Diet Study)

**DOI:** 10.1007/s00394-021-02753-3

**Published:** 2021-12-02

**Authors:** Stine Weder, Markus Keller, Morwenna Fischer, Katja Becker, Ute Alexy

**Affiliations:** 1 Research Institute for Plant-Based Nutrition, 35444 Biebertal, Germany; 2 Biochemistry and Molecular Biology, Interdisciplinary Research Center, Justus Liebig University Giessen, 35392 Giessen, Germany; 3 45525 Hattingen, Germany; 4 IEL-Nutritional Epidemiology, DONALD Study, University of Bonn, Heinstück 11, 44225 Dortmund, Germany

**Keywords:** Vegetarian, Vegan, Children, Critical nutrients, Micronutrients, Fatty acids

## Abstract

**Purpose:**

There is an ongoing debate whether vegetarian (VG) and especially vegan (VN) diets are nutritionally adequate in early childhood. Hence, the Vegetarian and Vegan Children Study (VeChi Diet Study) aimed to assess the food and nutrient intake of VG and VN infants.

**Methods:**

The study examined the diets of 1–3-year-old VG, VN, and omnivorous (OM) children (*n* = 430). Dietary intake was assessed via a 3-day weighed dietary record and compared between groups using ANCOVA. Lifestyle data were collected using a questionnaire. Here, the results of micronutrient and fatty acid intakes are presented.

**Results:**

Most nutrient intakes (with and without supplements) differed significantly between VN children and the two other groups, with a more favourable overall micronutrient intake in VN, followed by VG children, [e.g., the highest intake of vitamin E (8.3 mg/d vs. VG 7.4 mg/d and OM 5.1 mg/d), vitamin B_1_ (569 µg/d vs. VG 513 µg/d and OM 481 µg/d), folate (143 µg/d vs. VG 116 µg/d and OM 108 µg/d), magnesium (241 mg/d vs. VG 188 mg/d and OM 164 mg/d), and iron (8.9 mg/d vs. VG 7.3 mg/d and OM 6.0 mg/d)] as well as fat quality [highest intake of polyunsaturated fatty acids (8.7 E% vs. VG 6.9 E% and OM 4.5 E%) and lowest intake of saturated fatty acids (9.1 E% vs. VG 11.9 E% and OM 14.0 E%)]. In contrast, OM children had the highest intake of vitamin B_2_ (639 µg/d vs. VG 461 µg/d and VN 429 µg/d), calcium (445 mg/d vs. VG 399 mg/d and VN 320 mg/d), iodine (47 µg/d vs. VG 33 µg/d and VN 31 µg/d), and DHA (35.4 mg/d vs. VG 16.6 mg/d and VN 18.4 mg/d). Without supplementation, OM children had the highest average vitamin B_12_ intake (1.5 µg/d vs. VG 0.6 µg/d and VN 0.2 µg/d), whereas VN children had the highest average vitamin B_12_ intake with supplementation (73.8 µg/d vs. VG 1.3 µg/d and OM 1.7 µg/d). Without supplementation, none of the groups’ median intakes met the harmonised Average Requirement (h-AR) for vitamin D and iodine. Moreover, VG and VN children did not achieve h-ARs for vitamin B_2_, vitamin B_12_, and iron—if a low absorption of iron is anticipated; VN children also did not do so for calcium.

**Conclusion:**

In early childhood, VN and VG diets can provide most micronutrients in desirable amounts and a preferable fat quality compared to an OM diet. Special focus should be paid to (potentially) critical nutrients, particularly vitamin D, iodine, and DHA for all children regardless of diet, as well as vitamin B_2_, vitamin B_12_, calcium, and iron for VG and VN children.

**Trail registration:**

This study was registered with the German Clinical Trials Register (DRKS00010982) on (September 2, 2016).

**Supplementary Information:**

The online version contains supplementary material available at 10.1007/s00394-021-02753-3.

## Introduction

Vegetarian (VG, excluding meat and fish) and vegan (VN, excluding all animal foods) diets have become increasingly popular recently [1]. Nevertheless, only a few studies have investigated the food intake, nutritional status, and health of VG and VN children. These studies were heterogeneous, mostly cross-sectional, and of small sample sizes. Furthermore, they were mainly from the 1970s to 1990s [2, 3]. Since then, the food market has changed, for example, offering more dairy or meat alternatives and dietary supplements. Hence, new studies are needed to estimate the food and nutrient intake of children on a modern VG or VN diet.

Only four studies have examined the micronutrient intake of VG, VN, and OM children during the last 10 years [4–7]. Potentially critical micronutrients identified in these studies are calcium, sometimes iron, and vitamin D for VG children. However, they mostly had a higher intake of vitamin E, vitamin B_1_, vitamin C, and magnesium, lower intake of vitamin B_2_, vitamin B_12_ (without supplements), and zinc, and sometimes higher intake of (pro)vitamin A, and folate than OM subjects [4, 6, 8–16]. The intake of saturated fatty acids (SFA) [4, 5] and dietary cholesterol [5] of VG children was lower, and polyunsaturated fatty acid intake (PUFA) [4] was higher than in OM subjects.

VN children generally met the recommended intakes of essential nutrients in older studies [17, 18]. In one study, they did not reach the recommendations for vitamin B_12_ [17], in two studies those for vitamin D [18, 19], and in all three studies for calcium [17–19]. In the study by Sanders and Purves (1981), as well as in the follow-up in 1988 [19], a few VN children had a low intake of vitamin B_2_ and vitamin B_12_ [18]. VN children had a higher intake of linoleic acid (LA), a high ratio of LA: α-linolenic acid (ALA), and PUFA, but a lower intake of SFA than OM children [17]. None of these rather outdated studies examined the nutritional status of VN children with biomarkers, and the contribution of supplements to the nutrient intake was also not evaluated in detail. In more recent studies with VN children, they had higher intakes of vitamin E [4], β-carotene equivalents [6], vitamin B_1_ [4], vitamin C [4, 6], folate [4–6], magnesium [4, 6], zinc [4, 5], iron [4–6], MUFA [4, 5], and PUFA [4–6], but lower intakes of vitamin B_2_ [4], vitamin B_12_ (without supplements) [4, 6], vitamin D [6], calcium [4, 6], SFA [4–6], and dietary cholesterol [5, 6] than VG and/or OM children.

The nutritional status of VG children in most studies was generally normal [5, 9, 10, 14, 20, 21]. In some studies, the concentrations of vitamin B_12_ (without supplements) [6], vitamin D [11, 13], iron [9, 15, 21], or zinc [5] were lower than the cutoffs and/or concentrations in OM children. Regarding blood lipids, VG had lower average total cholesterol (C) [6, 22], LDL-C [22], HDL-C [6], but higher VLDL-C [6], and triglycerides [6, 22] than OM children. In other studies, the blood lipids did not differ in comparison to OM participants [4, 5] or were within the reference ranges [10].

Regarding nutritional status, VN children had higher folate concentrations [4, 5], but poorer status markers for iron [4, 6], vitamin A [5], vitamin D [5, 6], and vitamin B_12_ (without supplements) in some studies [6]. Regarding blood lipids, VN children had lower DHA [5], triglyceride [4], HDL-C [5, 6], non-HDL-C [4], LDL-C [4–6], and total C [5, 6] concentrations than VG and/or OM children. However, in the VeChi Youth Study, there were no significant differences in blood concentrations of haemoglobin between VN, VG, and OM participants. Nevertheless, VN subjects had lower ferritin concentrations than OM children, but mostly in the normal range. Furthermore, no significant differences in blood concentrations of vitamin B_2_ and 25-OH vitamin D were found between the groups. Additionally, VG but *not* VN participants on average had a poorer vitamin B_12_ status than OM subjects [4].

Dietary habits appear to vary by age group and country. Hence, the overall aim of the Vegetarian and Vegan Children Study (VeChi Diet Study) was to examine the food and nutrient intake of 430 VG, VN, and OM children aged 1–3 years in Germany. Results on anthropometric data and macronutrient intakes were published recently [23]. This paper describes micronutrient and fat intake and assesses the nutrient adequacy of the three diet groups.

## Subjects and methods

### Study population

The VeChi Diet Study is a cross-sectional study collecting data on diet, lifestyle, body weight (BW), and body height (BH) from VG, VN, and OM children. The study was conducted from October 2016 to April 2018 throughout Germany. Study design, sample size calculation, recruitment, and initial results are described elsewhere in detail [23]. In short, the study population consisted of 127 VG, 139 VN, and 164 OM children aged 1–3 years (Fig. S1, Online Resource 1). Families were recruited mainly via the study website (www.vechi-studie.de), VN/VG/child nutrition Facebook groups, a mailing list of Giessen University, magazines and journals, VN/VG websites, daycare centres, and VN/VG conventions. Furthermore, participating families were asked to recruit friends of their children. The subjects were without diagnosed diseases that could affect the studied variables. Moreover, diets with predominantly uncooked (raw) food (≥ 70%) were excluded.

The observational and non-invasive VeChi Diet Study was conducted according to the guidelines of the Declaration of Helsinki and its later amendments. The Ethics Committee of the University of Bonn approved it (046/17). All examinations were performed with parental written consent.

### Diet group classification

Parents were asked in an online questionnaire: “How is your child raised?”.

- Vegetarian (no meat, sausage, or fish, but with dairy products and/or eggs).
- Vegan (no meat, sausage, fish, dairy products, or eggs).
- Omnivorous (with meat and/or sausage and/or fish).

Parents of VG or VN children were asked whether there are exceptions in food intake. Accordingly, VG and VN children who usually eat meat or fish ≥ 1 time/week were reclassified as OM (2.1%: 8 VG, 1 VN). VN who usually eat dairy products and/or eggs ≥ 1 time/week were categorized as VG (5.6%: 24 VN).

### Dietary assessment

Dietary intake was assessed using weighed dietary records (paper-and-pencil) during 3 consecutive days (weekdays and weekends). The parents weighed and recorded all foods, beverages, and supplements that the participating children consumed, as well as plate waste, to the nearest gram using their own electronic kitchen scales. When exact weighing was not possible—e.g., when eating out—household measures (e.g., spoons, cups) and a photo booklet with foods in toddlers’ portion sizes [24], supplemented with special VG and VN foods, allowed semi-quantitative recording. Besides written information on dietary recording, a video tutorial was provided on the study website. The protocols were returned via mail or email. The study staff assessed missing data, requesting the information from the parents via email. When children ate at a daycare, preschool teachers recorded the foods the children ate, supplemented by recipes from the caterer if available. When recipes were not available, they were simulated. As measurement of individual breast milk intakes was not possible, amounts were estimated by multiplying the numbers of breast meals with age-specific amounts of breast milk meal volumes (for details see [23]). Energy and nutrient intakes were calculated using the nutrient food composition database LEBTAB. In this database, the composition of staple foods is based on the standard German food composition tables BLS 3.02. The energy and nutrient contents of commercial food products, i.e., processed foods and ready-to-eat meals or snack foods, were estimated via recipe simulation using labelled ingredients and nutrient contents [25]. LEBTAB is continuously updated by adding products or supplements recorded by study participants. Supplements were included in the calculation of individual nutrient intake with the exact composition and dosage as supplementation, in particular of vitamin B_12_, as recommended for vegans [26–29].

### Assessment of covariables

Data on sociodemographic variables, lifestyle, and further variables, e.g., birth weight, were collected via an online questionnaire. Reported BW and BH were either proxy assessed by the parents or by a paediatrician during the last paediatric checkup. Sex-specific weight-for-height, height-for-age, and weight-for-age z-scores were calculated using WHO Anthro version 3.2.2 for SPSS [30]. Socioeconomic status (SES) was calculated according to [31]. Physical activity was categorized into active or very active (i.e., ‘playing outside’ and/or ‘attendance in play/sport groups’ ≥ 4–7 times per week) or less active (< 4 times/week).

### Data analysis and statistics

Statistical analysis was performed with SPSS Version 20 (IBM SPSS Statistics, Chicago, IL, USA). Energy and nutrient intake (with and without dietary supplements) were calculated as individual means of the 3 recorded days (kcal, mg, or µg/d). Fortified foods, e.g., multivitamin juices, fortified ready-to-eat cereals, and plant oils fortified with DHA, were included in the food intake. Fatty acids, carbohydrates, and protein were additionally calculated as percentages of energy intake (E%). We used the harmonised Average Requirement (h-AR), a combination of the AR of the European Food Safety Authority (EFSA) and the Estimated AR (EAR) of the Institute of Medicine (IOM) of the US National Academies [32] to estimate the percentage of subjects at risk of nutrient deficiency. To reflect the different bioavailability, EFSA published two different AR values for iron for 1–3-year-old children: one for moderate absorption (5 mg/d) and one for low absorption (10 mg/d). The comparison of the medians with both AR values is presented. Nevertheless, we assume moderate absorption for an OM diet and low absorption for a VG and VN diet. Since no ARs for potassium, vitamin K, and fatty acids are available, only group comparisons were carried out.

Participants’ characteristics are presented as mean ± standard deviation (SD) for the variables with a normal distribution. For non-normally distributed variables, median and interquartile ranges (IQRs) are presented. Differences in categorical characteristics between diet groups were tested using Chi^2^ test or Fisher’s exact test. For continuous characteristics, ANOVA for parametric or the Kruskal–Wallis test for non-parametric data was applied. In the case of significant differences, pairwise Bonferroni post hoc tests (parametric) or Mann–Whitney *U* tests (non-parametric data) were applied.

Continuous variables were included in the analysis of covariance (ANCOVA) as covariates [children’s age (years), breast milk intake (g/day), paternal BMI (kg/m^2^), socioeconomic status [Winkler index], weight-for-height *z*-score]. Categorical variables were included as fixed factors (sex, physical activity, season). In case of unequal categorical variables (e.g., urbanicity), these variables were dummy coded. Total energy intake (TEI) was only considered as a confounder for variables that were not calculated as E%. In the basic model, sex and age were included. Then, each covariate was checked separately for interaction effects with sex and age. Those covariates or interactions with a *p* ≤ 0.1 and/or a partial eta squared (*η*^2^) ≥ 0.06 were added to the model. The backward method was used to build the fully adjusted model with the same criteria (*p* ≤ 0.1 and/or partial *η*^2^ ≥ 0.06). If heterogeneity of variances as an assumption of ANCOVA was violated for the fully adjusted model, simple bootstrap ANCOVA with 1,000 samples and bias-corrected accelerated (BCa) 95% confidence intervals (CI) were calculated.

Data transformations $\left( {\log \left[ x \right],\log \left[ {x + 1} \right],{\text{ square root}}\left[ x \right]} \right)$ were applied if assumptions of ANCOVA were violated. Due to a high number of tests, the chance of type I errors had to be reduced. Consequently, *p* ≤ 0.001 is considered statistically significant. An *η*^2^ ≥ 0.01 is interpreted as small, *η*^2^ ≥ 0.06 as medium, and *η*^2^ ≥ 0.14 as large effect sizes [33].

Sensitivity analyses without outliers (modulus of standardized residuals > 3) were carried out. Results are presented if there was remarkable variance from the main analysis.

Due to the skewness of the data and the resulting violation of assumptions, ANCOVA of vitamin B_12_ and vitamin D intake could not be conducted. Instead, the vitamin B_12_ and D intakes were adjusted for 4.184 MJ (= 1,000 kcal/day) and tested for differences with a Kruskal–Wallis and a Dunn–Bonferroni post hoc test. Effect size *r* is calculated with $r = \left| {\frac{z}{\sqrt n }} \right|$.

## Results

### Sample characteristics

Sample characteristics are presented in Table 1. The age of the study participants ranged from 0.9–4.3 years and 51.9% were boys (not shown). The majority of families lived in metropolitan or medium-sized urban cities, and the median SES was high. The proportion of children using any dietary supplement or vitamin B_12_ supplements was highest among VN children. The diet groups did not differ concerning most characteristics.

**Table 1:** Sample characteristics of the VeChi Diet Study by diet group

	VN	VG	OM
Total	139 (32.3)	127 (29.5)	164 (38.1)
Sex^†^			
Female	76 (54.7)	64 (50.4)	83 (50.3)
Male	63 (45.3)	63 (49.6)	81 (49.4)
Age years^a‡^	1.8 (1.3)	2.0 (1.4)	2.0 (1.5)
TEI MJ/d^a‡^	4.1 (1.5)	4.0 (1.2)	4.1 (1.0)
Carbohydrate intake E%^a‡^	53.8 (9.9)	53.6 (7.8)	53.1 (9.4)
Protein intake E%^a‡^	11.2 (2.5)^1^	11.1 (2.6)^2^	13.2 (2.7)^1,2^
Protein intake g/kg BW^a‡^	2.3 (0.9)^1^	2.3 (0.8)^2^	2.5 (0.9)^1,2^
Child body weight kg^b‡^	12.0 ± 2.5	12.1 ± 2.3	12.7 ± 2.6
Child body height cm^b‡^	85.6 ± 8.8	86.6 ± 8.8	88.2 ± 9.3
Weight-for-height *z*-score^b‡^	0.16 ± 1.08	0.11 ± 0.95	0.23 ± 0.96
Height-for-age *z*-score^b^^‡^	0.01 ± 1.26	0.11 ± 1.34	0.13 ± 1.01
Weight-for-age *z*-score^a^^‡^	0.11 ± 0.93	0.17 ± 0.99	0.25 ± 0.87
Urbanicity (*n* = 429)^†^			
Rural < 5000	28 (20.1)	19 (15.0)	28 (17.2)
Small-size urban 5000– < 20,000	20 (14.4)	16 (12.6)	17 (10.4)
Medium-size urban 20,000– < 100,000	31 (22.3)	33 (26.0)	31 (19.0)
Metropolitan ≥ 100,000	60 (43.2)	59 (46.5)	87 (53.4)
SES Winkler index score^†^			
Low 3–8	2 (1.4)	3 (2.4)	0 (0.0)
Middle 9–14	37 (26.6)	31 (24.4)	30 (18.3)
High 15–21	100 (71.9)	93 (73.2)	134 (81.7)
Physical activity (*n *= 429)^†^			
Less active < 4 times/week	70 (50.0)	71 (55.5)	64 (39.8)
Active or very active	70 (50.0)	57 (44.5)	97 (60.2)
Season of dietary record^†^			
Spring	45 (32.4)	21 (16.5)	36 (22.0)
Summer	17 (12.2)	20 (15.7)	38 (23.2)
Autumn	36 (25.9)	41 (32.3)	43 (26.2)
Winter	41 (29.5)	45 (35.4)	47 (28.7)
Paternal BMI (kg/m^2^) (*n *= 425)^a‡^	24.3 (4.1)	24.5 (3.9)	25.7 (3.1)
Breastfeeding			
Ever breastfed^†^	138 (99.3)	121 (95.3)	157 (95.7)
Duration of exclusively breastfeeding (mo)^a‡^	15.9 (10.0)^1, 2^	13.1 (10.0)^1^	11.1 (7.0)^2^
Breastfed during dietary record^†^	68 (48.9)^1^	34 (26.8) ^1^	17 (10.4)^1^
Breast milk intake in dietary record in kcal/d (all participants)^a, b^^‡^	0 (22.1)^1^ 13.4 ± 18.5^1^	0 (6.2)^1^ 7.2 ± 14.6^1^	0 (0.0)^1^ 2.3 ± 9.0^1^
Breast milk intake in dietary record (g/d) (only breastfed subjects, *n *= 119)^a‡^	303.3 (280.0)	275.0 (186.3)	163.3 (316.7)
Dietary supplement intake in dietary records, all supplements^†^			
Yes	135 (97.1)^1, 2^	68 (53.5)^1^	66 (40.2)^2^
No	4 (2.9)^1, 2^	59 (46.5)^1^	98 (59.8)^2^
Vitamin B_12_ supplement intake in dietary records^†^			
Yes	135 (97.1)^1^	45 (35.4)^1^	13 (7.9)^1^
No	4 (2.9)^1^	82 (64.6)^1^	151 (92.1)^1^
Dietary supplement intake in the online questionnaire but not in dietary records, all supplements^†^			
Yes	3 (2.2)^1^	13 (10.2)^2^	33 (20.1)^1,2^
Iodised salt use in general or daily use of iodised bread^†^			
Yes	90 (65.7)^1^	89 (70.1)^2^	141 (86.5)^1,2^
No	47 (34.3)^1^	38 (29.9)^2^	22 (13.5)^1,2^
Weekend days in the dietary records^†^			
0 weekend days	57 (41.0)	49 (38.6)	49 (29.9)
1 weekend day	37 (26.6)	33 (26.0)	50 (30.5)
2 weekend days	45 (32.4)	41 (32.2)	63 (38.4)
3 weekend days	0 (0.0)	4 (3.2)	2 (1.2)
Start of the diet^†^			
With the introduction of supplementary food	123 (88.5)	107 (84.3)	140 (85.4)
Later	16 (11.5)	18 (14.2)	24 (14.6)
Missing	0 (0.0)	2 (1.5)	0 (0.0)
Mothers’ diets^†^			
VN	136 (97.8)^1^	42 (33.1)^1^	10 (6.1)^1^
VG	3 (2.2)^1^	80 (63.0)^1^	16 (9.8)^1^
OM	0 (0.0)^1^	5 (3.9)^1^	138 (84.1)^1^

Unless indicated otherwise values are expressed in frequencies (percentages)

*VN* vegan, *VG* vegetarian, *OM* omnivorous, *TEI* total energy intake, *E%* % of energy intake, *BW* body weight, *SES* socioeconomic status, *BMI* body mass index

Differences of categorical variables were analysed using ^†^Chi^2^ test or Fisher’s exact test for cell frequencies of < 20% of expected counts less than 5

^‡^ANOVA and Bonferroni post hoc tests for parametric, Kruskal–Wallis and Mann–Whitney U tests for nonparametric, continuous data

^a^Values are median (IQR)

^b^Values are mean ± SD

^1,2^Values with the same superscripts are statistically different (at least *p* < 0.001)

### Micronutrient intake

Intakes (without supplements) of all vitamins except vitamin A (retinol eq) and vitamin D differed significantly between groups. VN children had the highest intake of vitamins E, K, B_1_, B_6_, folate, and C. In contrast, OM children had the highest intake of vitamins B_2_ and B_12_. Between VG and OM children, there were only significant differences in the intake of vitamins E, B_2_ and B_12_ (Table 2).

**Table 2:** Median daily intake (without supplements) of vitamins and minerals of vegan (VN), vegetarian (VG), and omnivorous (OM) children in the VeChi Diet Study by diet group

	VN (*n *= 139)	VG (*n *= 127)	OM (*n *= 164)	h-AR	Basic model^∆^		Fully adjusted model^‡^	
					*p*	Partial *η*^2^	*p*	Partial *η*^2^
Vitamin A (retinol eq) (µg/d)^a^	550 (377–779)	475 (331–654)	560 (372–854)	205	0.008	0.022	0.008	0.024
β-carotene (mg/d)^b^	3.2 (1.9–5.1)	2.5 (1.4–3.8)	2.3 (1.4–4.6)	–	0.020	0.018	0.002	0.031
Vitamin E (mg/d)^c^	8.3 (6.1–11.7)^1^	7.4 (5.1–9.9)^1^	5.1 (3.9–7.0)^1^	5.0	< 0.0001	0.200	< 0.0001	0.196^§^
Vitamin K (µg/d)^d^	82 (53–120)^1,2^	67 (41–86)^1^	46 (26–72)^2^	–	< 0.0001	0.099	< 0.0001	0.110
Vitamin B_1_ (µg/d)^e^	569 (437–754)^1,2^	513 (377–611)^1^	481 (398–605)^2^	400	< 0.0001	0.038	< 0.0001	0.124
Vitamin B_2_ (µg/d)^f^	429 (325–537)^1^	461 (375–641)^2^	639 (517–800)^1,2^	500	< 0.0001	0.175	< 0.0001	0.202^§^
Vitamin B_6_ (mg/d)^g^	0.8 (0.6–1.1)^1,2^	0.7 (0.6–0.8)^1^	0.7 (0.6–0.9)^2^	0.5	0.002	0.030	< 0.0001	0.117
Folate (µg/d)^h^	143 (106–197)^1,2^	116 (96–149)^1^	108 (90–135)^2^	90	< 0.0001	0.148	< 0.0001	0.148^§^
Vitamin C (mg/d)^i^	63 (44–84)^1^	54 (41–66)	45 (32–63)^1^	15	< 0.0001	0.076	< 0.0001	0.073^§^
Potassium (mg/d)^j^	1839 (1387–2204)^1,2^	1567 (1227–1858)^1^	1513 (1309–1861)^2^	–	< 0.0001	0.065	< 0.0001	0.113^§^
Calcium (mg/d)^k #^	320 (251–453)^1^	399 (280–567)	445 (356–553)^1^	390	< 0.0001	0.059	< 0.0001	0.060
Magnesium (mg/d)^l^	241 (180–310)^1^	188 (143–240)^1^	164 (134–195)^1^	65	< 0.0001	0.147	< 0.0001	0.292^§^
Iron (mg/d)^m^	8.9 (6.0–11.6)^1^	7.3 (5.5–9.0)^1^	6.0 (4.7–7.4)^1^	5.0/10.0*	< 0.0001	0.111	< 0.0001	0.300
Zinc (mg/d)^n^	4.9 (3.7–6.2)	4.7 (3.8–5.6)	5.0 (4.1–5.8)	3.6	0.194	0.008	0.111	0.012
Iodine (µg/d)^o^	31 (22–44)^1^	33 (23–45)^1^	47 (36–61)^1^	65	< 0.0001	0.118	< 0.0001	0.167^§^

	VN (*n *= 139)	VG (*n *= 127)	OM (*n *= 164)	h-AR	*p *†	r		
						VN vs VG	VG vs OM	VN vs OM
Vitamin B_12_ (µg/d)	0.2 (0.1–0.4)^1^	0.6 (0.3–1.0)^1^	1.5 (1.1–2.3)^1^	0.7	< 0.0001	0.399	0.471	0.656
Vitamin D (µg/d)	0.7 (0.3–1.1)	0.8 (0.4–1.4)	0.8 (0.5–1.6)	10	0.006	0.120	0.017	0.143

Values are presented as median (25th–75th percentile)

*VN* vegan, *VG* vegetarian, *OM* omnivorous, *DRV* dietary reference value, *eq* equivalents, *BMI* body mass index, *SES* socioeconomic status

*h-AR for moderate absorption of iron (5.0 mg/d) vs. low absorption (10.0 mg/d)

^#^Without outliers (*n *= 4)

^§^Partial *η*^2^ was higher without outliers

^∆^*p* values and effect sizes were derived from ANCOVA adjusted for age and sex

^‡^*p* values and effect sizes were derived from ANCOVA adjusted for age, sex, and other confounders (see ^a−o^)

^†^*p* values and effect sizes were calculated with Kruskal–Wallis and Dunn–Bonferroni post hoc tests adjusted for the energy intake (µg/4.184 MJ/d)

^a^Fully adjusted model (square root transformed) adjusted for age, sex, physical activity, SES, energy intake, paternal BMI (*n *= 424)

^b^Fully adjusted model (square root transformed) adjusted for age, sex, breast milk intake, weight-for-height *z*-score, SES, energy intake, season (*n *= 430)

^c^Fully adjusted model (ln transformed) adjusted for age, sex, breast milk intake, weight-for-height *z*-score, SES, seasons (*n *= 430)

^d^Fully adjusted model (ln transformed) adjusted for age, sex, breast milk intake, SES, energy intake, season (*n *= 430)

^e^Fully adjusted model (ln transformed) adjusted for age, sex, breast milk intake, energy intake, season, urbanicity (*n *= 429)

^f^Fully adjusted model (ln transformed) adjusted for age, sex, breast milk intake, SES, energy intake, paternal BMI (*n *= 425)

^g^Fully adjusted model (ln transformed) adjusted for age, sex, breast milk intake, energy intake (*n *= 430)

^h^Fully adjusted model (ln transformed) adjusted for age, sex, breast milk intake, energy intake, paternal BMI, urbanicity (*n *= 424)

^i^Fully adjusted model (ln transformed) adjusted for age, sex, energy intake, season (*n *= 430)

^j^Fully adjusted model adjusted for age, sex, breast milk intake, energy intake, seasons (*n *= 430)

^k^Fully adjusted model adjusted for age, sex, breast milk intake, energy intake, physical activity, SES, seasons (*n *= 429)

^l^Fully adjusted model (ln transformed) adjusted for age, sex, breast milk intake, energy intake, weight-for-height *z*-score, paternal BMI, seasons (*n *= 425)

^m^Fully adjusted model (ln transformed) adjusted for age, sex, breast milk intake, energy intake, weight-for-height *z*-score, physical activity, SES, seasons, urbanicity (*n *= 428)

^n^Fully adjusted model (ln transformed) adjusted for age, sex, breast milk intake, energy intake, SES, paternal BMI, urbanicity, seasons (*n *= 422)

^o^Fully adjusted model (ln transformed) adjusted for age, sex, breast milk intake, energy intake, SES, paternal BMI (*n *= 425)

^1,2^Values with the same superscripts are statistically different in the fully adjusted model (Bonferroni adjusted, at least *p* < 0.001)

For minerals (Table 2), intakes (without supplements) of all minerals except zinc differed significantly between groups. VN children showed the highest intake of potassium, magnesium, and iron, whereas OM children had the highest intake of calcium and iodine. Between VG and OM children, only magnesium, iron, and iodine intake differed significantly.

When supplements were taken into account, vitamins B_12_ and D intake differed significantly between VN and VG, as well as VN and OM children (Table 3). The differences between the groups’ average nutrient intake were the same if supplements were included; only the effect sizes were usually greater without supplements. Table S1 (Online Resource 1) additionally shows the mean intake of vitamins, minerals, and long-chain n-3 fatty acids obtained from food and dietary supplements.

**Table 3:** Median daily intake (with supplements) of vitamins, minerals, and long-chain n3-fatty acids of vegan (VN), vegetarian (VG), and omnivorous (OM) children in the VeChi Diet Study by diet group

	VN (*n *= 139)	VG (*n *= 127)	OM (*n *= 164)	h-AR		Basic model^∆^		Fully adjusted model^‡^	
						*p*	Partial *η*^2^	*p*	Partial *η*^2^
Vitamin A (retinol eq) (µg/d)^a^	554 (381–794)	475 (331–676)	570 (372–854)	205	0.008		0.023	0.006	0.025
β-carotene (mg/d)^b^	3.3 (1.9–5.1)	2.5 (1.4–3.9)	2.3 (1.4–4.6)	–	0.019		0.019	0.002	0.031
Vitamin E (mg/d)^c^	8.3 (6.1–11.4)^1^	7.4 (5.1–9.7)^1^	5.1 (3.9–7.0)^1^	5.0	< 0.0001		0.175	< 0.0001	0.169^§^
Vitamin K (µg/d)^d^	87 (52–128)^1,2^	66 (39–86)^1^	47 (26–73)^2^	–	< 0.0001		0.103	< 0.0001	0.117
Vitamin B_1_ (µg/d)^e^	572 (448–789)^1,2^	514 (377–625)^1^	481 (398–610)^2^	400	< 0.0001		0.037	< 0.0001	0.094^§^
Vitamin B_2_ (µg/d)^f^	448 (336–591)^1^	478 (386–673)^2^	639 (523–822)^1,2^	500	< 0.0001		0.080	< 0.0001	0.073^§^
Vitamin B_6_ (mg/d)^g^	0.9 (0.6–1.2)^1,2^	0.7 (0.6–0.9)^1^	0.7 (0.6–0.9)^2^	0.5	< 0.0001		0.048	< 0.0001	0.107^§^
Folate (µg/d)^h^	153 (113–218)^1,2^	117 (98–151)^1^	108 (91–138)^2^	90	< 0.0001		0.129	< 0.0001	0.156^§^
Vitamin C (mg/d)^i^	63 (47–89)^1^	56 (41–68)	47 (32–64)^1^	15	< 0.0001		0.078	< 0.0001	0.070^§^
Potassium (mg/d)^j^	1839 (1387–2204)^1,2^	1568 (1227–1858)^1^	1513 (1321–1868)^2^	–	< 0.0001		0.064	< 0.0001	0.112^§^
Calcium (mg/d)^k #^	327 (251–467)^1^	392 (280–567)	452 (357–564)^1^	390	< 0.0001		0.054	< 0.0001	0.055
Magnesium (mg/d)^l^	244 (181–310)^1^	189 (143–240)^1^	164 (135–195)^1^	65	< 0.0001		0.184	< 0.0001	0.346
Iron (mg/d)^m^	9.2 (6.1–11.9)^1^	7.3 (5.5–9.0)^1^	6.0 (4.7–7.4)^1^	5.0/10.0*	< 0.0001		0.125	< 0.0001	0.272^§^
Zinc (mg/d)^n^	5.0 (3.6–6.3)	4.8 (3.8–5.7)	5.0 (4.1–5.8)	3.6	0.185		0.008	0.028	0.019
Iodine (µg/d)^o^	34 (23–48)	33 (23–45)	48 (36–63)	65	< 0.0001		0.082	< 0.0001	0.082§
20:5*n*-3 (EPA) (mg/d)^p^	4.4 (1.0–9.3)^1^	1.6 (0.4–5.4)^2^	10.7 (4.3–46.6)^1,2^	–	< 0.0001		0.229	< 0.0001	0.250^§^
22:6*n*-3 (DHA) (mg/d)^q^	19.1 (6.2–42.6)^1^	19.5 (6.8–37.9)^2^	35.4 (15.6–82.3)^1,2^	–	< 0.0001		0.088	< 0.0001	0.158^§^

	VN (*n *= 139)	VG (*n *= 127)	OM (*n *= 164)	h-AR	*p*^†^	*r*	
						VN vs VG	VN vs OM
Vitamin B_12_ (µg/d)	71.4 (5.5–333.5)^1,2^	1.3 (0.6–6.1)^1^	1.6 (1.1–2.6)^2^	0.7	< 0.0001	0.366	0.521
Vitamin D (µg/d)	12.9 (3.7–25.1)^1,2^	2.1 (0.6–13.0)^1^	1.7 (0.6–12.9)^2^	10	< 0.0001	0.230	0.305

Values are presented as median (25th–75th percentile)

*VN* vegan, *VG* vegetarian, *OM* omnivorous, *DRV* dietary reference value, *eq* equivalents, *EPA* eicosapentaenoic acid, *DHA* docosahexaenoic acid, *BMI* body mass index, *SES* socioeconomic status

*h-AR for moderate absorption of iron (5.0 mg/d) vs. low absorption (10.0 mg/d)

^‡^*p* values and effect sizes were derived from ANCOVA adjusted for age, sex and other confounders (see ^a−q^)

^†^*p* values and effect sizes were calculated with Kruskal–Wallis and Dunn–Bonferroni post hoc test adjusted for the energy intake (µg/4.184 MJ/d)

^§^Partial *η*^2^ was higher without outliers

^#^Without outliers

^∆^*p* values and effect sizes were derived from ANCOVA adjusted for age and sex

^a^Fully adjusted model (square root transformed) adjusted for age, sex, physical activity, SES, energy intake, paternal BMI (*n *= 424)

^b^Fully adjusted model (square root transformed) adjusted for age, sex, breast milk intake, weight-for-height *z*-score, SES, energy intake, season (*n *= 430)

^c^Fully adjusted model (ln transformed) adjusted for age, sex, breast milk intake, weight-for-height *z*-score, SES, seasons (*n *= 430)

^d^Fully adjusted model (ln transformed) adjusted for age, sex, breast milk intake, SES, energy intake, season (*n *= 430)

^e^Fully adjusted model (ln transformed) adjusted for age, sex, breast milk intake, energy intake, season, urbanicity (*n *= 429)

^f^Fully adjusted model (ln transformed) adjusted for age, sex, breast milk intake, SES, energy intake, paternal BMI (*n *= 425)

^g^Fully adjusted model (ln transformed) adjusted for age, sex, breast milk intake, energy intake (*n *= 430)

^h^Fully adjusted model (ln transformed) adjusted for age, sex, breast milk intake, energy intake, paternal BMI, urbanicity (*n *= 424)

^i^Fully adjusted model (ln transformed) adjusted for age, sex, energy intake, season (*n *= 430)

^j^ Fully adjusted model adjusted for age, sex, breast milk intake, energy intake, seasons (*n *= 430)

^k^Fully adjusted model adjusted for age, sex, breast milk intake, energy intake, physical activity, SES, seasons (*n *= 429)

^l^Fully adjusted model (ln transformed) adjusted for age, sex, breast milk intake, energy intake, weight-for-height z-score, paternal BMI, seasons (*n *= 425)

^m^Fully adjusted model (ln transformed) adjusted for age, sex, breast milk intake, energy intake, weight-for-height *z*-score, physical activity, SES, seasons, urbanicity (*n *= 428)

^n^Fully adjusted model (ln transformed) adjusted for age, sex, breast milk intake, energy intake, SES, paternal BMI, urbanicity, seasons (*n *= 422)

^o^Fully adjusted model (ln transformed) adjusted for age, sex, breast milk intake, energy intake, SES, paternal BMI (*n *= 425)

^p^Fully adjusted model (log transformed) adjusted for age, sex, breast milk intake, physical activity, energy intake, seasons (*n *= 429)

^q^Fully adjusted model (log transformed) adjusted for age, sex, breast milk intake, physical activity, energy intake, seasons (*n *= 429)

^1,2^ Values with the same superscripts are statistically different in the fully adjusted model (Bonferroni adjusted, at least *p* < 0.001)

Fig. S2 (Online Resource 1) shows the intake of vitamins and minerals (without supplements) as percentages of the h-ARs [32] (%h-AR) by diet group. In all diet groups, median %h-AR was below 100% for vitamin D and iodine, as well as for iron if a low absorption is anticipated. Median %h-AR reached or was above 100% for vitamins A, E, B_1_, B_6_, C, folate, magnesium, zinc, and iron if a moderate absorption is anticipated. For vitamin B_12_, VN children clearly fell below the h-AR when supplements were not considered. Less than half of VN children reached the h-AR for calcium and vitamin B_2_, whereas the median intake of OM children reached the h-AR of both nutrients, while the median intake of VG children was between the other two groups (below the h-AR for vitamins B_2_ and B_12_, above the h-AR for calcium).

### Fatty acid and cholesterol intake

The intake of SFA, PUFA, LA, ALA, DHA, and cholesterol differed significantly between all groups in the fully adjusted model with large effect sizes (Table 4). DHA and EPA intake with supplements differed significantly between VN and OM, as well as VG and OM children, respectively (Table 3). Without supplements, the difference in DHA intake was also significant between VG and VN children (Table 4). OM children had the highest intake of SFA, arachidonic acid (AA), EPA, DHA, and cholesterol. In contrast, VN children had the highest intake of PUFA, LA, and ALA.

**Table 4:** Median daily intake (without supplements) of fatty acids and cholesterol of vegan (VN), vegetarian (VG), and omnivorous (OM) children in the VeChi Diet Study by diet group

	VN (*n *= 139)	VG (*n *= 127)	OM (*n *= 164)	Basic model^∆^		Fully adjusted model^‡^	
				*p*	Partial *η*^2^	*p*	Partial *η*^2^
Total fat (E%)^a^	33.6 (27.9–39.4)^1^	33.7 (29.7–36.6)^1^	32.6 (28.2–37.2)^1^	0.781	0.001	< 0.0001	0.049
Total SFA (E%)^b^	9.1 (6.4–12.6)^1^	11.9 (9.5–15.1)^1^	14.0 (11.6–17.4)^1^	< 0.0001	0.199	< 0.0001	0.261
Total MUFA (E%)^c^	12.1 (9.6–14.9)	11.3 (9.3–13.4)	10.9 (9.2–12.6)	0.011	0.021	0.903	0.001
Total PUFA (E%)^d^	8.7 (7.2–10.5)^1^	6.9 (5.3–8.5)^1^	4.5 (3.7–5.6)^1^	< 0.0001	0.431	< 0.0001	0.389^§^
18:2*n*-6 (LA) (E%)^e^	7.0 (6.1–8.1)^1^	5.8 (4.4–6.9)^1^	3.6 (3.0–4.6)^1^	< 0.0001	0.420	< 0.0001	0.384^§^
18:3*n*-3 (ALA) (E%)^f^	1.0 (0.7–2.0)^1^	0.7 (0.5–1.2)^1^	0.6 (0.4–0.8)^1^	< 0.001	0.184	< 0.0001	0.185
LA:ALA^g^	7.0 (3.9–9.9)	6.7 (4.6–9.5)	6.3 (4.8–7.8)	0.070	0.013	0.018	0.020
20:4*n*-6 (AA) (mg/d)^h^	7.2 (2.6–16.3)^1^	12.0 (5.4–21.7)^1^	34.3 (21.9–54.6)^1^	< 0.0001	0.370	< 0.0001	0.514^§^
20:5*n*-3 (EPA) (mg/d)^i^	3.8 (0.9–8.6)^1^	1.4 (0.4–5.2)^2^	10.7 (4.3–46.5)^1,2^	< 0.0001	0.316	< 0.0001	0.349
22:6*n*-3 (DHA) (mg/d)^j^	18.4 (6.0–38.3)^1^	16.6 (6.0–30.9)^1^	35.4 (15.6–82.2)^1^	< 0.0001	0.133	< 0.0001	0.222^§^
Cholesterol (mg/4.184 MJ/d)^k^	15.0 (0.3–76.3)^1^	54.0 (20.4–117.4)^1^	91.8 (58.9–145.3)^1^	< 0.0001	0.237	< 0.0001	0.493

Values are presented as median (25th–75th percentile)

*VN* vegan, *VG* vegetarian, *OM* omnivorous, *CI* confidence interval, *E%* % of energy intake, *SFA* saturated fatty acids, *MUFA* monounsaturated fatty acids, *PUFA* polyunsaturated fatty acids, *LA* linoleic acid, *ALA* α-linolenic acid, *AA* arachidonic acid, *EPA* eicosapentaenoic acid, *DHA* docosahexaenoic acid, *SES* socioeconomic status

^∆^*p* values and effect sizes were derived from ANCOVA adjusted for age and sex

^‡^*p* values and effect sizes were derived from ANCOVA adjusted for age, sex and other confounders (see ^a−k^)

^§^Partial *η*^2^ was higher without outliers

^a^Fully adjusted model adjusted for age, sex, breast milk intake, urbanicity (*n *= 429)

^b^Fully adjusted model adjusted for age, sex, breast milk intake, physical activity, paternal BMI, urbanicity, seasons (*n *= 423)

^c^Fully adjusted model adjusted for age, sex, breast milk intake, weight-for-height z-score, urbanicity (*n *= 429)

^d^Fully adjusted model adjusted for age, sex, breast milk intake, paternal BMI, seasons (*n *= 425)

^e^Fully adjusted model adjusted for age, sex, breast milk intake, paternal BMI, seasons (*n *= 425)

^f^Fully adjusted model (log transformed, using bootstrap) adjusted for age, sex, breast milk intake, paternal BMI (*n *= 425)

^g^Fully adjusted model (using bootstrap) adjusted for age, sex, breast milk intake, physical activity, paternal BMI, weight-for-height *z*-score (*n *= 424)

^h^Fully adjusted model (log transformed) adjusted for age, sex, breast milk intake, weight-for-height *z*-score, energy intake, seasons (*n* = 430)

^i^Fully adjusted model (log transformed) adjusted for age, sex, breast milk intake, physical activity, energy intake, seasons (*n* = 429)

^j^Fully adjusted model (log transformed) adjusted for age, sex, breast milk intake, physical activity, energy intake, seasons (*n* = 429)

^k^Fully adjusted model (square root transformed, using bootstrap) adjusted for age, sex, breast milk intake, physical activity (*n *= 429)

^1,2^Values with the same superscripts are statistically different in the fully adjusted model (Bonferroni adjusted, at least *p* < 0.001)

## Discussion

### Micronutrient intake

In our study, VN children had the highest intake of several vitamins and minerals. In contrast, OM children had the highest intake of vitamin B_2_, calcium, and iodine. Intake of vitamin D and iodine was below the h-AR in all groups. Only the median intake of vitamins B_2_ and B_12_ as well as iron by VG and VN, and calcium by VN children, was below the h-AR.

Our results confirm that, in VN diets, vitamin B_12_ supplementation is essential to ensure sufficient intake as bioactive vitamin B_12_ only occurs in animal foods [34, 35]. The proportion of VN children who received vitamin B_12_ supplements was very high. However, the remaining VN children are at risk of a deficiency. Apart from supplements, other sources of vitamin B_12_ are fortified products. Indeed, a recent regional survey of food retailers in Germany revealed that a variety of vegan foods are fortified with vitamin B_12_ [36]. Nevertheless, regarding the usual amounts of these food products consumed, vitamin B_12_ levels are generally too low to meet h-AR. It is important to note that the median vitamin B_12_ intake of VG children was also below the h-AR [32]. In a recent review, as well as in the VeChi Youth Study, a higher risk of vitamin B_12_ deficiency not only for VN, but also for VG was described [4, 35]. Hence, both VN and VG children should be encouraged to use vitamin B_12_ supplements on a regular and reliable basis and should be screened for vitamin B_12_ deficiency. In contrast, the median vitamin B_12_ intake of OM children seemed to be adequate.

Dairy products are the main source of vitamin B_2_ and calcium in Germany [37]. Not only VN but also VG children consumed less of this food group than OM children (data not shown and [38]). In our study, the intake of vitamin B_2_ and calcium was substantially lower among VN and VG than among OM children. However, only the median intake of VN children did not reach the h-AR of calcium, whereas the median intake of VG children just reached the h-AR. Therefore, vitamin B_2_ supplementation or consumption of vitamin B_2_-fortified foods might be recommended for all VG and VN children. In contrast, the VeChi Youth Study showed a high prevalence (> 35%) of vitamin B_2_ blood concentrations below the reference value regardless of the diet group [4].

The average intake of vitamin D (without supplementation) of all groups was also inadequate in our study. Calcium and vitamin D are key nutrients to build and maintain a normal bone mass [39]. In a recent systematic review and meta-analysis of 20 cohort and cross-sectional studies (*n *= 37,134), VG and VN adults had lower bone mineral densities and VN a higher fracture rate than OM adults [39]. However, this meta-analysis has been criticized due to the lack of statistical adjustment in particular of the BMI [40, 41]. Our results are mostly in agreement with two cross-sectional studies from Poland (*n *= 50, 2–10 years and *n *= 187, 5–10 years) where VG and VN children had calcium and vitamin D intakes below the reference values (only without vitamin D supplementation in [6]). More relevantly, 25-OH vitamin D blood levels were lower and the concentration of serum bone metabolism markers was poorer than those of OM children [6, 13]. In the study by Desmond et al. [6], VG and VN subjects had less bone mineral content than OM subjects, which was more pronounced for VN children. Supplementation of vitamin D resolved low 25-OH vitamin D concentrations. Likewise, the VeChi Youth Study showed a high prevalence (> 30%) of 25-OH vitamin D in all diet groups being below the reference value [4]. However, Hovinen et al. [5] showed only borderline sufficient vitamin D concentrations in all VN participants. In another recent study from Poland with 53 VG and 53 OM children, there were no significant differences in calcium or vitamin D intake, as well as 25-OH vitamin D blood levels (80% of both groups included vitamin D supplements). Furthermore, the groups did not differ regarding the absolute values of bone mineral density, but VG children had on average lower total and lumbar spine BMD z-scores [7]. This is in accordance with the VeChi Youth Study [4]. Therefore, a higher intake of calcium should be encouraged for VN children and perhaps also for VG children since the median intake of the latter only just reached the h-AR. Whether vitamin D supplementation is necessary beyond infancy is under discussion in Germany [42, 43].

Iodine intake was inadequate in our study in all diet groups. Major dietary sources of iodine in the diet of children in Germany are iodised salt and dairy [44]. Salt was not recorded quantitatively in our dietary records; hence, intakes presented here might be underestimated. However, this bias should not differ between the groups. In our study, OM children had the highest average iodine intake, due to the highest consumption of dairy and fish (data not shown), but also of iodised salt and foods, in particular bread, produced with iodised salt (Table 1). This is in line with the results of the VeChi Youth Study where VN children and adolescents had significantly lower urinary iodine excretion than OM and VG subjects. However, the excretion of all groups was on average below the reference value [45]. As iodine intake during childhood is associated with cognitive development, there is an urgent need for increased intake, for example via broader use of iodised salt for food production, in particular bread [46]. Alternatively, on an individual basis, iodine can be supplemented as recommended for breastfed infants in Germany without iodine-fortified weaning food [47, 48].

VN and VG diets are regarded as risk factors for iron deficiency [26, 49]. Nevertheless, available studies on iron deficiencies among VG and VN children and adolescents yielded heterogeneous results [50]. For example, Desmond et al. [6] found a higher prevalence of iron deficiency anaemia in VN children, whereas Hovinen et al. [5] found no differences between VN and OM children in serum ferritin. The ferritin concentrations in the VeChi Youth Study were significantly higher in OM participants than in VG and VN ones. Nevertheless, the prevalence of haemoglobin and ferritin concentrations below the cutoff was low [4]. As in other studies [4–6], VN and VG children in our study had higher iron intakes than OM children. The observed high iron intake reflects the high iron content of some plant foods, e.g., whole grain or legumes. Nevertheless, the bioavailability of this non-heme iron is lower than that of heme iron from meat. Besides, the absorption of non-heme iron is dependent on promoting factors (e.g., organic acids such as ascorbic acid) and impairing factors (e.g., phytate, polyphenols) [34, 49]. Food preparation, i.e., leavening, fermentation, and soaking of legumes and grains, reduces the content of phytate and improves iron absorption [49]. Moreover, some ‘partial physiologic adaptive responses in the absorption of non-heme iron are discussed for VG and VN. Besides, this cannot be verified for our participants with the current study design, which did not allow measurement of the bioavailability of nutrients. However, if we assume moderate absorption for an OM diet and low absorption for a VG and VN diet, VG and VN children are below the h-AR for low absorption and OM children are above the h-AR for moderate absorption. On this basis, higher iron intake should be encouraged only in VG and VN children.

Plant foods are the main sources of folate in the diet [51]. As a result, the median folate intake of VN was significantly higher than that of VG and OM children. The higher folate intake of VN children is in line with other recent studies [4–6]. However, all groups met the h-AR on average.

### Fatty acids and cholesterol intake

Altogether, the fat quality of the diet of VN children was the most favourable, followed by that of VG children; the VN children’s intake of MUFA and PUFA was highest and intake of SFA was lowest. Only OM children exceeded on average the recommended maximum daily intake of dietary cholesterol [80 mg/1000 kcal (4.184 MJ)] for children [43].

In contrast, the EPA and DHA intake of VN and VG were substantially lower than those of OM children. In OM diets, fatty fish is the major source for preformed DHA. The long-chain n-3 fatty acid is especially important in early childhood. Besides others, it plays a central role in eye and brain development in childhood [52]. For VG or VN diets, plant supplements from microalgae (e.g., *Schizochytrium sp.*) with DHA (+ EPA) are available, e.g., as fortified plant oils. The endogenous formation of DHA is low because the conversion rate of ALA into DHA is poor. However, it might be increased by replacing LA with ALA in the diet because both fatty acids compete for the same enzymes [52]. There was a trend to a higher LA:ALA ratio from the OM to the VG to the VN diet group. To improve the LA:ALA ratio, the consumption of good ALA sources (i.e., linseed oil, rapeseed oil) are recommended, especially in VG and VN children where sources of preformed EPA and DHA are limited.

### Strengths and limitations

Our study has some limitations. The cross-sectional design of the VeChi Diet Study allows no conclusion on the long-term food intake of the children. Nevertheless, follow-up investigations are planned. Furthermore, our data are based on proxy reporting by the parents (or the family paediatrician in case of BW and BH). Hence, the anthropometric data are especially susceptible to bias (discussed in [23]) [53, 54]. However, we used weighed dietary records because they provide the best estimate for children aged 0.5–4 years [55]. Parents were instructed to maintain the usual diet, and every protocol was checked for completeness and plausibility. Missing information was immediately collected from the parents. As seen in other investigations, underreporting is unlikely (only 1%) in 1–5-year-old children [56]. Other studies such as the DONALD study [57] have been using this method successfully for many years. Nevertheless, the data have not been confirmed with objective biomarkers. Nutrient intake was compared to the h-AR. Unfortunately, there are no h-ARs for vitamin K, potassium, and fatty acids. Furthermore, in general, the data basis to derive DRVs for children is sparse and ARs do not take the special requirements of a VN diet into account, except for iron and zinc.

The online questionnaire was not validated, but the questions were partly taken from a questionnaire of a representative, validated health survey in Germany (German Health Interview and Examination Survey for Children and Adolescents) [58], complemented by VG and VN-specific questions. Another limitation is the estimation of breast milk intake, which is based on reliable data of the DONALD study but is not as exact as weighing the children before and after each breastfeeding. Additionally, the mothers’ supplementation was not gathered. Moreover, the composition of breast milk of VN and VG women might vary from that of OM women. Additionally, three days of dietary record might not be enough to observe foods that are not consumed daily such as fish. Therefore, the DHA and iodine intake of OM children could be higher than reported here. Since 3 days of dietary records might also not be sufficient to represent regular supplement intake, this question was also asked in the online questionnaire. The number of participants who reported taking a supplement in the online questionnaire but not in the dietary record was highest among the OM children. Because the number of VN and VG children in Germany is not known, it was not possible to select a representative sample. Additionally, the OM control group does not represent the average German population of that age. As in all nutrition studies, participants tend to be more health conscious than the average population. The majority of the families lived in medium-sized urban or metropolitan cities and had a high median SES. As a consequence, the differences between the diet groups might have been even higher when compared to the average population than to the health-conscious control group in our study.

However, a major strength of the VeChi Diet Study is the detailed dietary record that is well suited for young children and the assessment of special food groups of VN and VG diets (e.g., meat and dairy alternatives). The nutrient database LEBTAB ensures high accuracy in nutrient intake due to brand-specific estimations of ingredients and nutrient contents by recipe simulation including fortified foods and supplements. Therefore, the LEBTAB database is more accurate when assessing the food intake of VG and VN than the national database BLS. Therefore, the VeChi Diet Study comprises a unique data pool of the understudied population of VG and VN children at the age of 1–3 years. For the future, repeated examinations of this cohort, preferably complemented with biomarkers of the nutritional status and measurements of child development, are in preparation.

## Conclusion

In the VeChi Diet Study, VN and VG children had a more favourable intake of several micronutrients and fatty acids than that of OM children, regardless of the intake of dietary supplements. Critical nutrients for all three diet groups were vitamin D (without supplements), iodine, and DHA, with OM children having the highest intakes. For VG and VN children, vitamins B_2_ and B_12_, and iron are considered critical, as well as calcium for VN children. Therefore, for VG and VN children foods rich in vitamin B_2_ (e.g., yeast, nuts, legumes), iron (in combination with foods that increase the bioavailability of iron), vitamin B_2_-fortified plant-based dairy alternatives, and iodised salt are recommended. Furthermore, DHA supplementation should be encouraged, in addition to reliable supplementation of vitamin B_12_ and possibly vitamin B_2_. For VN children, calcium-fortified plant-based dairy alternatives and mineral water with high calcium content can improve calcium intake. OM children should consume more PUFA, fatty fish or more ecological alternatives, DHA supplements (from microalgae), and less cholesterol and SFA containing animal foods. Supplementation of vitamin D should be considered in all diet groups, especially in autumn and winter. To evaluate the health and nutritional status of VN and VG children, our data should be complemented with biomarkers and longitudinal studies of VN and VG diets during growth.

## Supplementary Information

Below is the link to the electronic supplementary material.

Supplementary file1 (PDF 452 KB)

## Data Availability

Not applicable.
