# Supplementary material for: Intake of micronutrients and fatty acids of vegetarian, vegan, and omnivorous children (1–3 years) in Germany (VeChi Diet Study)
Source: Eur J Nutr. 2021 Dec 2;61(3):1507–20. doi: 10.1007/s00394-021-02753-3 (PMC8921058; doi:10.1007/s00394-021-02753-3)
Supplement: Supplementary file 1 — Supplementary file1 (PDF 452 KB) [file 394_2021_2753_MOESM1_ESM.pdf]

Intake of Micronutrients and Fatty Acids of Vegetarian, Vegan, and Omnivorous Children (1-3 Years) in Germany (VeChi Diet Study)

Stine Weder[1,2], Markus Keller[1], Morwenna Fischer[3], Katja Becker[2], Ute Alexy[4]

[1]Research Institute for Plant-Based Nutrition, 35444 Biebertal, Germany, ORCID: S. Weder 0000-0001-5360-1940, M. Keller 0000-0001-5789-6280;

[2]Biochemistry and Molecular Biology, Interdisciplinary Research Center, Justus Liebig University Giessen, 35392 Giessen, Germany, ORCID: 0000-0003-4673-3675;

[3] 45525 Hattingen, Germany;

[4]Corresponding author: IEL-Nutritional Epidemiology, DONALD Study, University of Bonn, Heinstück 11, 44225 Dortmund, Germany, E-Mail: alexy@uni-bonn.de, Tel.: +49-231-79221016, ORCID: 0000-0002-1488-5175.

Summary

This supplementary information includes a flow chart of the recruitment of the VeChi Diet Study (Fig. S1). Fig. S2 shows the intake of vitamins and minerals as percentages of the harmonised Average Requirement (%h-AR). Table S1 presents the mean intake of vitamins, minerals, and long chain n-3 fatty acids obtained from food and dietary supplements.

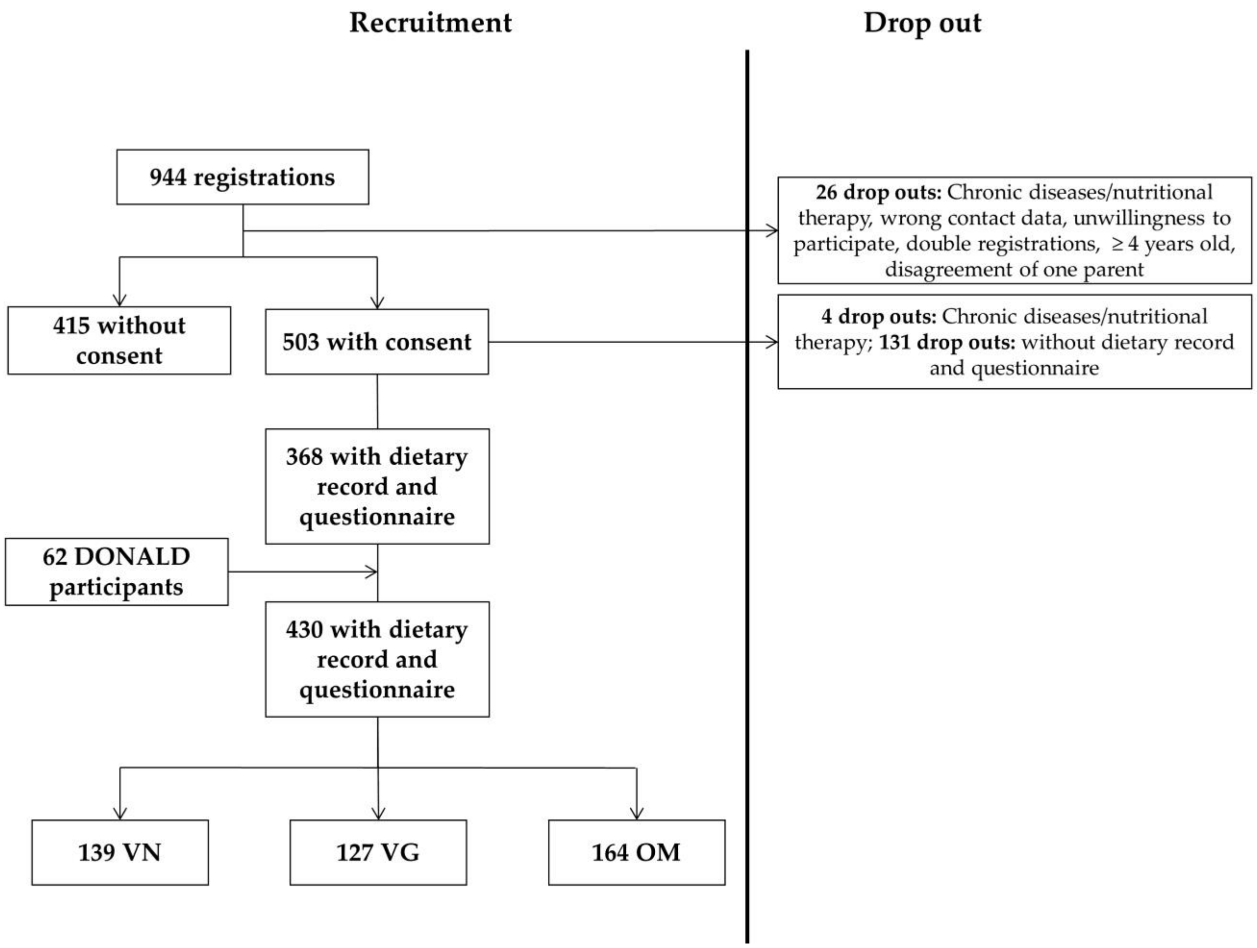

Fig. S1 Flow chart showing the recruitment of vegetarian (VG), vegan (VN), and omnivorous (OM) children in the VeChi Diet Study

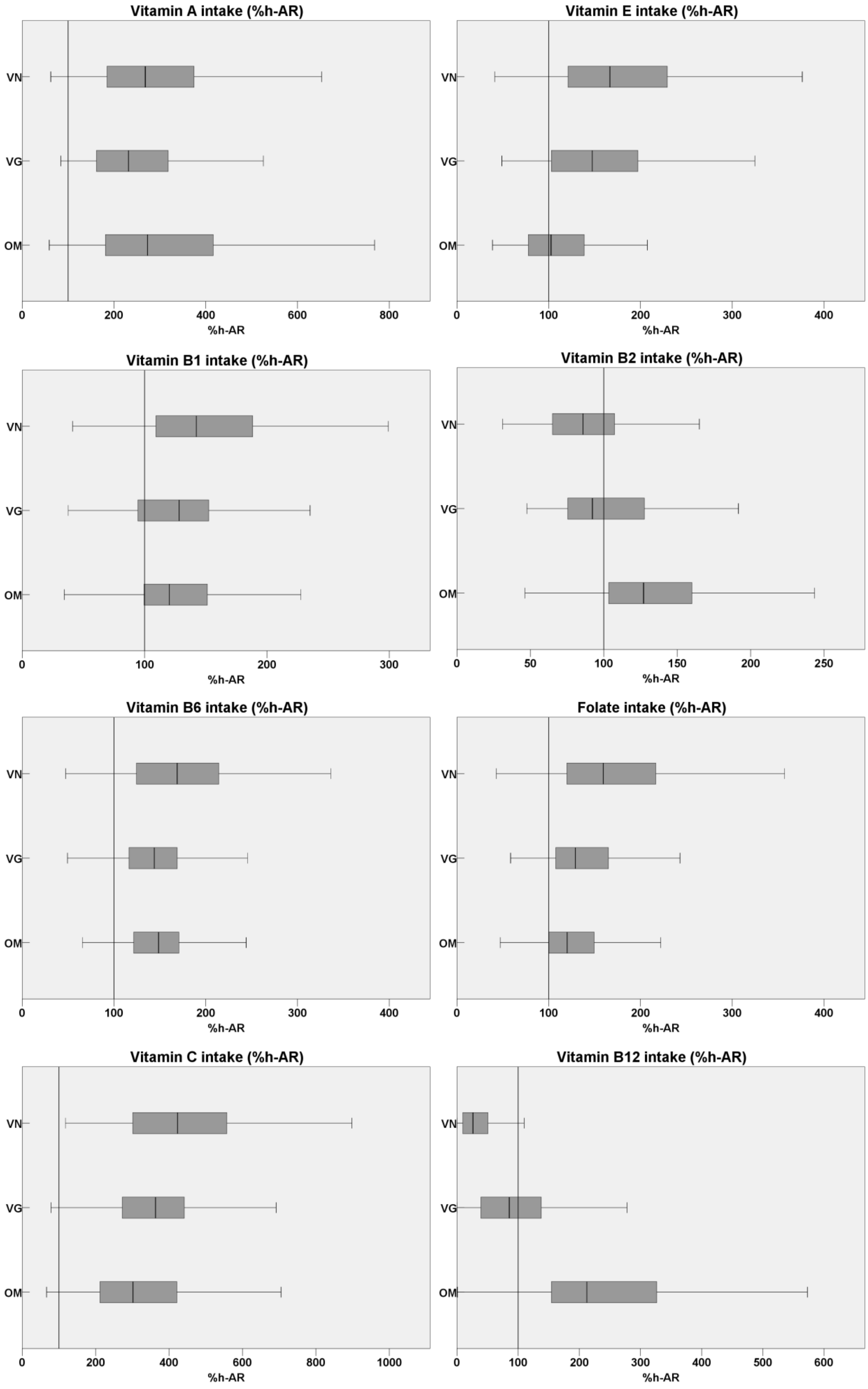
Vitamin A intake (%h-AR)
VN
VG
OM
0
200
400
600
800
%h-AR
Vitamin E intake (%h-AR)
VN
VG
OM
0
100
200
300
400
%h-AR
Vitamin B1 intake (%h-AR)
VN
VG
OM
0
100
200
300
%h-AR
Vitamin B2 intake (%h-AR)
VN
VG
OM
0
50
100
150
200
250
%h-AR
Vitamin B6 intake (%h-AR)
VN
VG
OM
0
100
200
300
400
%h-AR
Folate intake (%h-AR)
VN
VG
OM
0
100
200
300
400
%h-AR
Vitamin C intake (%h-AR)
VN
VG
OM
0
200
400
600
800
1000
%h-AR
Vitamin B12 intake (%h-AR)
VN
VG
OM
0
100
200
300
400
500
600
%h-AR

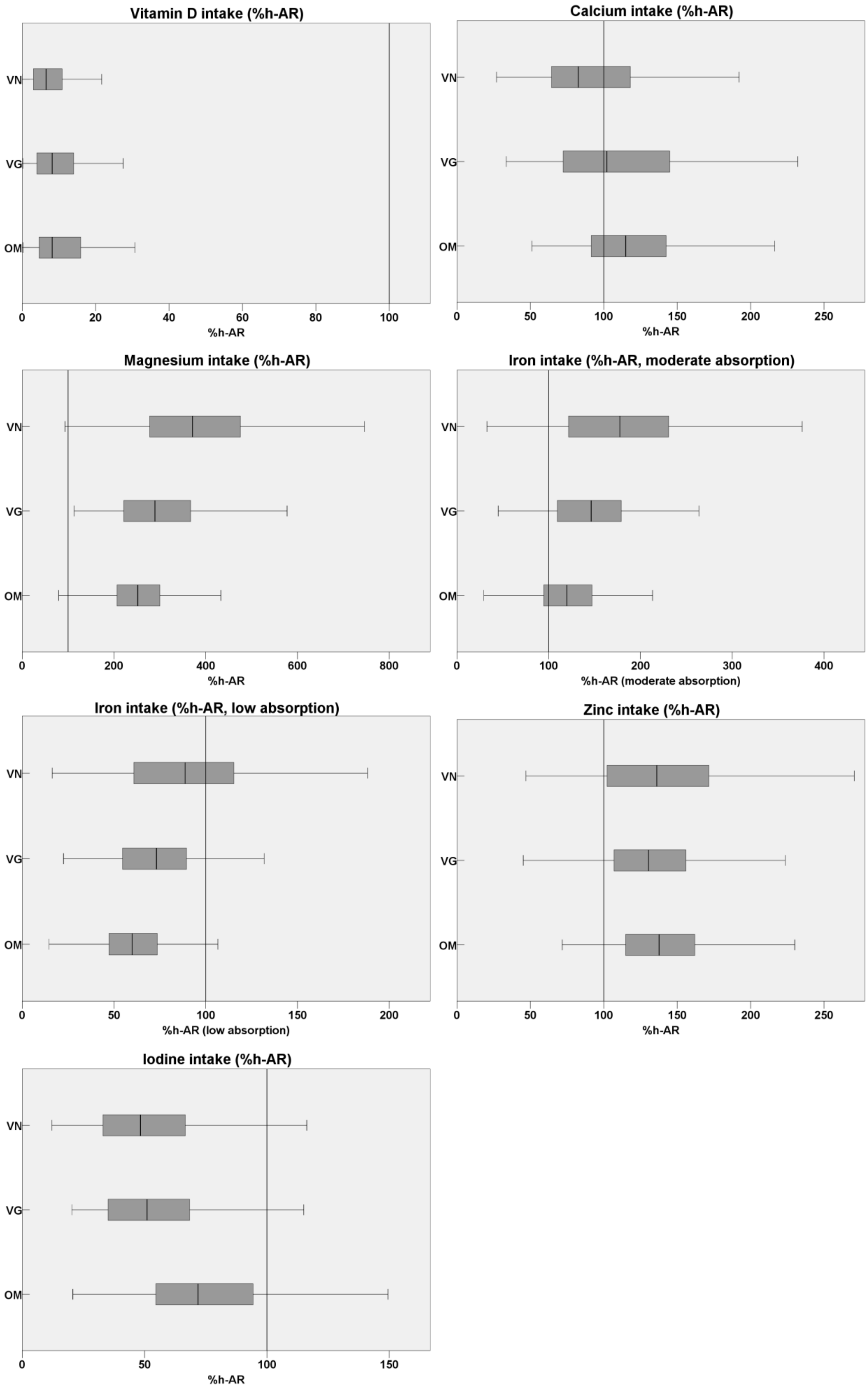
Vitamin D intake (%h-AR)
VN
VG
OM
0 20 40 60 80 100
%h-AR
Calcium intake (%h-AR)
VN
VG
OM
0 50 100 150 200 250
%h-AR
Magnesium intake (%h-AR)
VN
VG
OM
0 200 400 600 800
%h-AR
Iron intake (%h-AR, moderate absorption)
VN
VG
OM
0 100 200 300 400
%h-AR (moderate absorption)
Iron intake (%h-AR, low absorption)
VN
VG
OM
0 50 100 150 200
%h-AR (low absorption)
Zinc intake (%h-AR)
VN
VG
OM
0 50 100 150 200 250
%h-AR
Iodine intake (%h-AR)
VN
VG
OM
0 50 100 150
%h-AR

Fig. S2 Intake of vitamins and minerals as percentages of the harmonised Average Requirement (h-AR) (1) by diet group in 1–3-year-old children in the VeChi Diet Study. %h-AR, percentages of the harmonised Average Requirement; VN, vegan; VG, vegetarian; OM, omnivorous. Median [—], interquartile range [box; 25th and 75th percentiles], minimum and maximum value

Table S1 Mean intake of vitamins, minerals, and long chain n-3 fatty acids obtained from food and dietary supplements of vegan (VN), vegetarian (VG), and omnivorous (OM) children in the VeChi Diet Study

	VN (n = 139)		VG (n = 127)		OM (n = 164)		h-AR (1)
	**Food**	**Supplements**	**Food**	**Supplements**	**Food**	**Supplements**	
Vitamin A (retinol eq) (µg/d)	623 ± 361	16 ± 87	544 ± 324	3 ± 23	692 ± 517	3 ± 34	205
Vitamin E (mg/d)	9.2 ± 4.3	0.7 ± 3.2	7.8 ± 3.4	0.5 ± 3.8	5.7 ± 2.6	0.5 ± 5.4	5.0
Vitamin D (µg/d)	1 ± 1	19 ± 45	1 ± 1	6 ± 8	1 ± 1	5 ± 8	10
Vitamin K (µg/d)	98 ± 74	4 ± 15	71 ± 44	0 ± 3	57 ± 43	1 ± 7	-
Vitamin B_1 (µg/d)	605 ± 252	56 ± 253	512 ± 189	54 ± 321	513 ± 158	37 ± 405	400
Vitamin B_2 (µg/d)	469 ± 224	145 ± 504	525 ± 222	52 ± 266	678 ± 237	35 ± 284	500
Vitamin B_6 (mg/d)	0.9 ± 0.4	0.2 ± 0.6	0.7 ± 0.3	0.06 ± 0.4	0.8 ± 0.2	0.06 ± 0.5	0.5
Folate (µg/d)	160 ± 79	23 ± 69	125 ± 42	10 ± 73	116 ± 40	8 ± 90	90
Vitamin C (mg/d)	68 ± 31	7 ± 31	57 ± 24	4 ± 29	50 ± 24	4 ± 40	15
Vitamin B_{12} (µg/d)	0.3 ± 0.3	208 ± 305	0.8 ± 0.7	68 ± 177	1.8 ± 1.1	20 ± 147	0.7
Potassium (mg/d)	1842 ± 658	0 ± 2	1581 ± 486	0 ± 2	1591 ± 433	1 ± 4	-
Calcium (mg/d) [#]	368 ± 167	6 ± 34	428 ± 184	1 ± 11	471 ± 154	1 ± 10	390
Magnesium (mg/d)	250 ± 103	4 ± 27	197 ± 77	1 ± 6	171 ± 50	1 ± 8	65
Iron (mg/d)	8.9 ± 3.8	1.7 ± 7.0	7.4 ± 2.9	0.1 ± 0.5	6.2 ± 2.2	0.2 ± 1.4	moderate absorption: 5.0<br>low absorption: 10.0
Zinc (mg/d)	5.1 ± 1.9	0.3 ± 1.2	4.8 ± 1.6	0.1 ± 0.5	5.1 ± 1.3	0.1 ± 0.7	3.6
Iodine (µg/d)	35 ± 25	9 ± 26	44 ± 90	1 ± 8	50 ± 23	2 ± 8	65
20:5*n*-3 (EPA, mg/d)	5.6 ± 6.7	10.2 ± 43.7	3.4 ± 4.5	8.2 ± 42.0	35.6 ± 52.6	0 ± 0	-
22:6*n*-3 (DHA, mg/d)	27.5 ± 31.0	20.7 ± 79.7	21.3 ± 17.5	16.2 ± 73.0	65.5 ± 80.8	0 ± 0	-

VN, vegan; VG, vegetarian; OM, omnivorous; h-AR, harmonized Average Requirement; eq, equivalents.
Values are presented mean ± SD.
[#] without outliers (n = 4)

Literature Cited

1. Allen LH, Carriquiry AL, Murphy SP. Perspective: Proposed Harmonized Nutrient Reference Values for Populations. Adv Nutr 2020; 11(3):469–83.
